# Sex-dependent alterations in behavior, drug responses and dopamine transporter expression in heterozygous DAT-Cre mice

**DOI:** 10.1038/s41598-021-82600-x

**Published:** 2021-02-08

**Authors:** Kauê Machado Costa, Daniela Schenkel, Jochen Roeper

**Affiliations:** 1 Institute of Neurophysiology, Goethe-University Frankfurt, Theodor-Stern-Kai 7, 60596 Frankfurt am Main, Germany; 2 Present Address: National Institute on Drug Abuse Intramural Research Program, National Institutes of Health, 251 Bayview Blvd, Baltimore, MD 21224 USA

**Keywords:** Genetic techniques, Learning and memory, Basal ganglia, Reward

## Abstract

Heterozygous mice that express Cre-recombinase under the dopamine transporter promoter (DAT-Cre knock in mice, or KI) are widely used for targeting midbrain dopamine neurons, under the assumption that their constitutive physiology is not affected. We report here that these mice display striking sex-dependent behavioral and molecular differences in relation to wildtypes (WT). Male and female KI mice were constitutively hyperactive, and male KI mice showed attenuated hyperlocomotor responses to amphetamine. In contrast, female KIs displayed a marked reduction in locomotion (“calming” effect) in response to the same dose of amphetamine. Furthermore, male and female DAT-Cre KI mice showed opposing differences in reinforcement learning, with females showing faster conditioning and males showing slower extinction. Other behavioral variables, including working memory and novelty preference, were not changed compared to WT. These effects were paralleled by differences in striatal DAT expression that disproportionately affected female KI mice. Our findings reveal clear limitations of the DAT-Cre line that must be considered when using this model.

## Introduction

Transgenic Cre recombinase-expressing mouse lines are ubiquitous tools for genetically targeting specific cell types^1–3^. This is based on the Cre/LoxP system, which combines the expression of Cre with the flanking of target genes with LoxP sites (floxing) to either remove (conditional knock-out, or KO) or insert (conditional knock-in, or KI) particular genomic sequences^4–6^. Mouse lines where the Cre gene is inserted under the control of a cell-specific promoter are widely used for achieving conditional mutagenesis. They are combined with the introduction, usually through viral methods or crossbreeding, of floxed sequences containing an exogenous gene or of LoxP sites flanking a target genomic sequence. In addition to cell type-specific targeting, the Cre/LoxP system allows for the induction of gene changes during adulthood, circumventing developmental issues potentially present in conventional KO or KI mice^3,6^. Cre lines are often used under the implicit assumption that the insertion and expression of the Cre gene produces no, or a “minimal”, phenotype per se, especially in behavioral studies^3,7^. The typical interpretation of studies using this system is that observed effects are attributable to the unfloxing and subsequent expression or removal of the gene of interest^2,7^. It is therefore not only critical to provide evidence that Cre expression is indeed restricted to the desired target cell type^8^, but also that Cre gene insertion itself does not affect gene expression, at least in heterozygous animals (as monoallelic Cre expression is sufficient for conditional mutagenesis).

However, this is not always the case. In addition to the potential of Cre-induced cellular toxicity^9,10^, the insertion of the Cre gene often leads to under- or over-expression of genes under the control of the same promoter, and thus to significant physiological and behavioral alterations^11–14^. For example, Chen et al.^15^ recently demonstrated that two choline acetyltransferase (ChAT)-Cre lines, used to target cholinergic neurons, show altered behaviors related to cholinergic function, including baseline locomotion, responses to nicotine and operant food training.

Given that such inadvertent phenotypes might confound experimental results, we performed a detailed behavioral characterization of heterozygous transgenic mice where Cre is expressed under the dopamine transporter (DAT) promoter i.e. DAT-Cre KI mice. This line is widely used for targeting midbrain dopamine neurons. Importantly, we took into account sex as a biological variables and explicitly tested both male and female mice, following current guidelines on the matter^16–19^. These mice were derived from the first DAT-Cre line, where the Cre gene was inserted in the 5′ untranslated region of the DAT locus^20^. This is one the most used lines in the dopamine research field, and has recently been shown to be superior for dopamine research compared mice where Cre is expressed under the tyrosine hydroxylase promoter, where there is substantial off-target expression^8^. We report here that heterozygous DAT-Cre KI mice exhibit sex-specific differences in several dopamine-sensitive behaviors which is paralleled by differences in striatal DAT expression and discuss the implications of these findings for the use of this line.

## Results

### DAT-Cre KI mice have sex-dependent changes in baseline and amphetamine-induced locomotor activity

**Figure 1:**
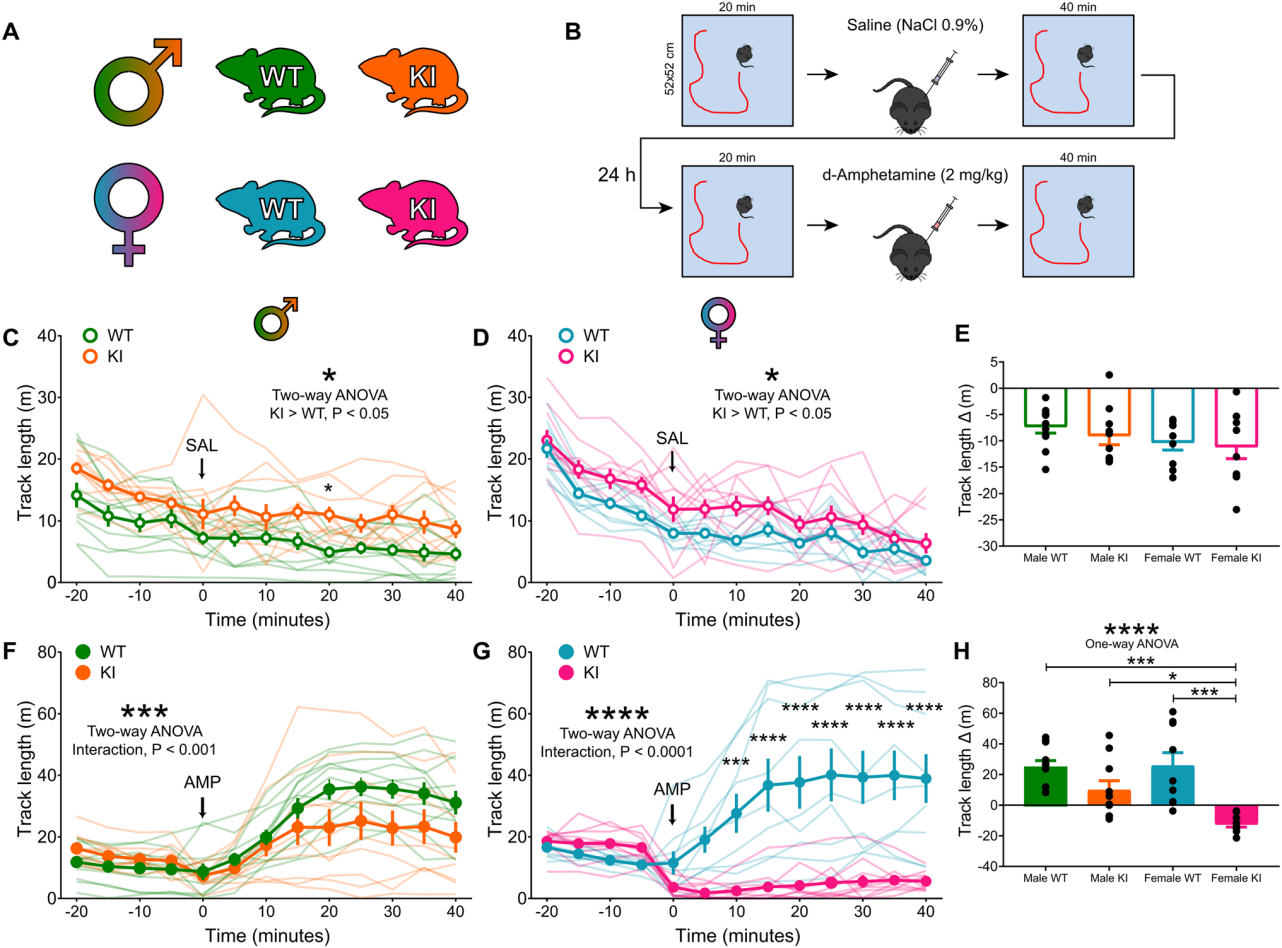
DAT-Cre KI mice have sex-dependent changes in baseline and amphetamine-induced locomotor activity. (**A**) Mice were divided according to their genotype and sex. The color-coding scheme illustrated here applies to all other figures in this study. (**B**) Mice were placed in an open field for 20 min, given a saline injection, and returned to the arena for 40 min. The next day animals went through a similar procedure, except they received injections of d-amphetamine (2 mg/kg). (**C**) Total distance travelled (5-min bins) for male WT and KI mice. KI mice displayed a hyperlocomotor phenotype. Note that saline injection creates only a minimal disruption in behavior. (**D**) Same as (**C**), but for females. Note that female KIs were also hyperactive. (**E**) Difference in distance travelled between the last and first 20 min of the experiment. There was no significant difference between groups. (**F**) Locomotor activity of male mice before and after d-amphetamine injections. Male KIs showed a blunted response to the drug. (**G**) Same as E, but for females. While WT females show increases in open field locomotion after the amphetamine injections, KI females decreased their activity. (**H**) Same as (**E**), but for the amphetamine experiment. Note that most groups showed an average increase in locomotion, except for the female KIs, which showed a consistent reduction in activity. Asterisks atop specific data points indicate multiple comparison tests results between groups. *P < 0.05; **P < 0.01; ***P < 0.001; ****P < 0.0001.

DAT-Cre KI and WT mice displayed striking sex-dependent differences in several dopamine-sensitive behaviors. First, male and female KI mice were constitutively hyperactive in the open field test (male genotype effect, KI > WT, *F*_1, 19_ = 8.03, *P* = 0.01*; female genotype effect, KI > WT, *F*_1, 17_ = 7.42, *P* = 0.014*), covering on average over 35% and 45% more of the track length covered by WT littermates during one hour of testing, respectively (Fig. 1C,D). The absence of an interaction effect between genotype and time for both sexes (female interaction effect, *F*_12, 204_ = 0.64, *P* = 0.8; male interaction effect, *F*_12, 228_ = 0.51, *P* = 0.91) suggests that the dynamics of open field habituation was not different in KI compared to WT mice, despite the higher levels of locomotor activity, which was confirmed by comparing the difference in locomotion between the first and last 20 min epochs of testing (Fig. 1E). Importantly, saline injections did not cause major changes in locomotor behavior in the open field in any of the tested groups (Fig. 1C,D,E).

When tested for amphetamine-induced hyperlocomotion, male KI had a slight, but consistent, attenuation of the locomotor effect in relation to WTs (interaction effect, *F*_12, 28_ = 3.19, *P* = 0.0003***) following amphetamine (2 mg/kg i.p.) injections (Fig. 1F). On average this effected amounted to a near 35% reduction in locomotion. Female KI mice, on the other hand, were strikingly different from their WT counterparts in their response to amphetamine (genotype effect, WT > KI, *F*_1, 17_ = 15.93, *P* = 0.0009***; interaction effect, *F*_12, 204_ = 17.45, *P* < 0.0001****). Female KI mice did not display any increased locomotor activity; indeed, they showed a marked decrease in covered track length immediately after amphetamine injections that persisted throughout the session (Fig. 1G). This effect was qualitatively different to all other groups (Fig. 1H). Overall, mean locomotor activity in the female WT group after amphetamine injections was nearly *eight* times higher than those in the KI group.

### DAT-Cre KI mice exhibit sex-dependent differences in reinforcement learning

**Figure 2:**
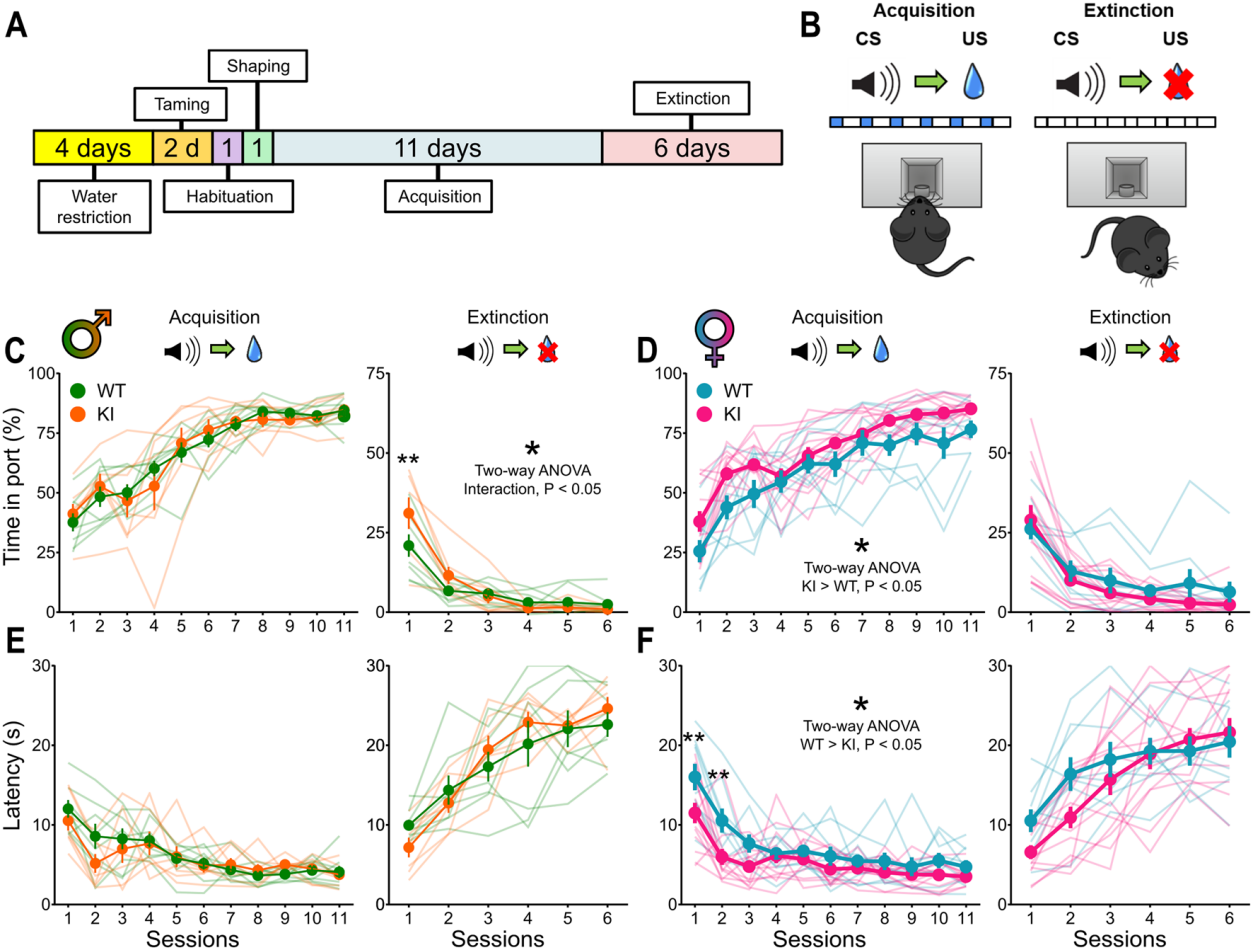
DAT-Cre KI mice have sex-dependent alterations in reinforcement learning. (**A**) Timeline of reinforcement learning experiment. (**B**) Cartoon of outcome contingencies and expected behaviors in the task. During acquisition, an auditory cue signals the availability of water reward, that is delivered in cycles as long as the mouse keeps its head in the port. In extinction, the same cue is presented, but no water is delivered. (**C**,**E**) Time in port (**C**) and latency to respond (**E**) during the acquisition and extinction of a conditioned response in male KI and WT mice. Male KI mice were comparable to controls during acquisition, but were slower to extinguish responding, as measured by the time in port measure. (**D**,**F**) Same as (**C**) and (**E**), but for female mice. Female KI mice, in contrast to males, were similar to controls during extinction, but responded more and faster to the cues throughout the entire acquisition phase, especially in the initial sessions, as was better revealed in the latency measure. Asterisks atop individual data points indicate multiple comparison results between groups. *P < 0.05; **P < 0.01.

In a reinforcement learning task, where mice learned to associate an auditory cue with the availability of sucrose water reward, we found that male KI and WT mice had similar learning during acquisition training (Fig. 2A,C) as measured by the time in port during cue presentation (genotype effect, *F*_1, 13_ = 0.001, *P* = 0.993; interaction effect, *F*_10, 130_ = 0.671, *P* = 0.75) and latency to respond (genotype effect, *F*_1, 13_ = 0.331, *P* = 0.575; interaction effect, *F*_10, 130_ = 1.029, *P* = 0.423). However, male KI mice showed significantly delayed extinction learning in relation to WT mice, as measured by the time in port (interaction effect, *F*_5, 65_ = 3.269, *P* = 0.011*; genotype effect, *F*_1, 13_ = 0.742, *P* = 0.405), but not in latency (genotype effect, *F*_1, 13_ = 0.076, *P* = 0.787; interaction effect, *F*_5, 65_ = 1.349, *P* = 0.255). Female KI mice, on the other hand, differed from WTs during acquisition learning, but not in extinction (Fig. 2D,F). Female KI mice spent more time within the reward port during the cue (genotype effect, KI > WT, *F*_1, 20_ = 5.45, *P* = 0.03*) but with similar learning dynamics (interaction effect, *F*_10, 200_ = 1.108, *P* = 0.357). This was also true for the latency (genotype effect, WT > KI, *F*_1, 20_ = 7.626, *P* = 0.012*; interaction effect, *F*_10, 200_ = 1.712, *P* = 0.08) as female KI mice responded faster to the cue throughout the acquisition sessions. During extinction, female KI and WT mice did not differ in time spent in port (genotype effect, *F*_1, 20_ = 0.944, *P* = 0.343; interaction effect, *F*_5, 100_ = 1.052, *P* = 0.392) or in latency, despite a very strong trend towards an interaction effect (genotype effect, *F*_1, 20_ = 0.764, *P* = 0.392; interaction effect, *F*_10, 200_ = 2.3, *P* = 0.0504).

### Other dopamine-dependent behaviors were not affected by DAT-Cre KI

**Figure 3:**
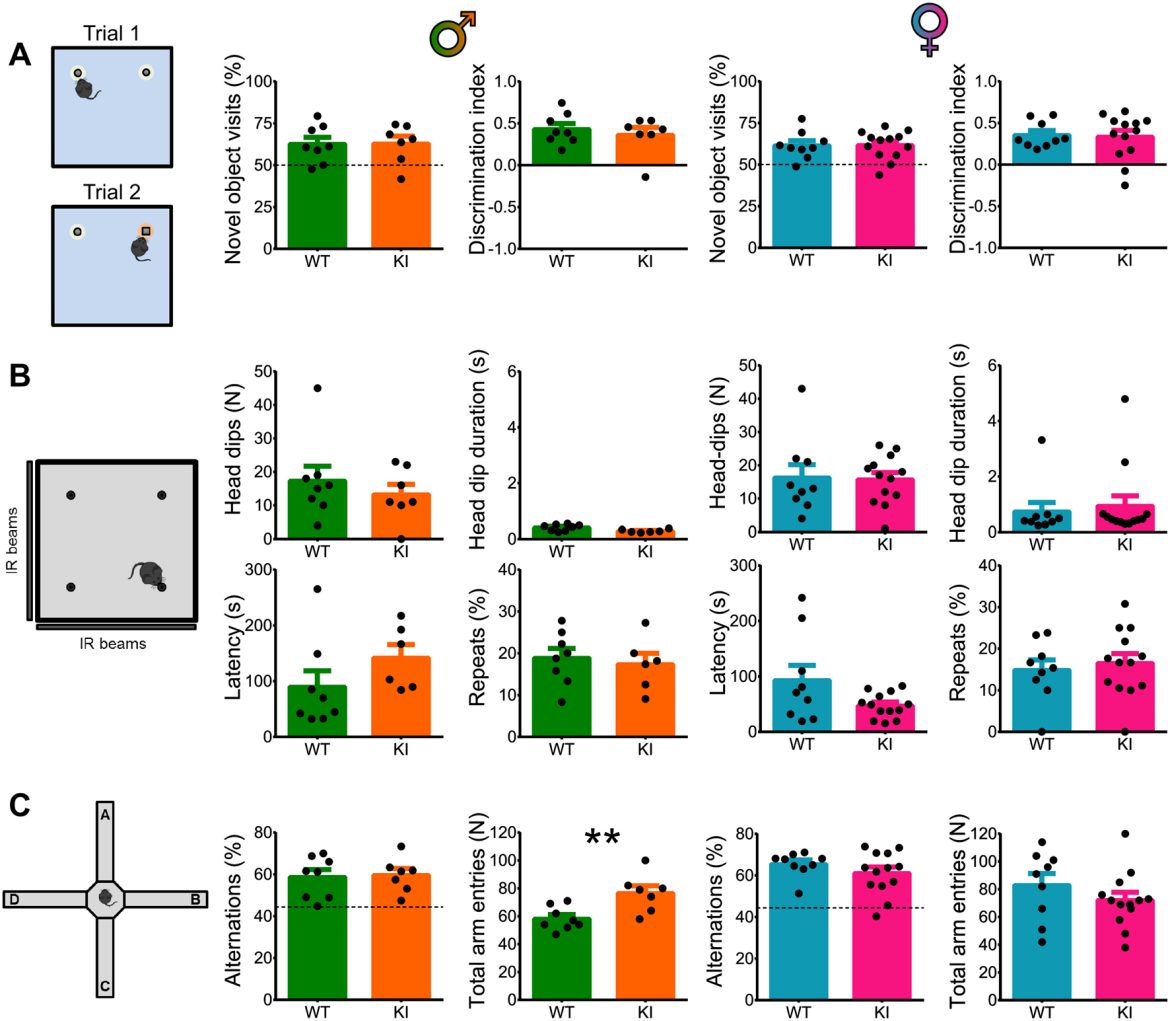
DAT-Cre KI does not affect novel object preference, hole-board exploration or working memory. (**A**) Results of the novel object preference test in KI and WT mice of both sexes. Learning is demonstrated by both the percentage of visits to the novel object in Trial 2 (in relation to all object visits), and by the discrimination index (ratio of time spent exploring the novel versus the familiar object). Both genotypes performed equally regardless of sex. (**B**) Measures of exploratory activity in the hole-board arena, including the number of head dips, average dip duration, latency to the first dip and the percentage of repeat dips. No difference between genotypes was found in both sexes. (**C**) Performance in the plus maze. Working memory in this task is measured by the percentage of spontaneous alternations in arm entries during maze exploration. All groups showed a similar percentage of spontaneous alternations. Note that male KI mice performed more arm entries (higher activity) than WT controls but had the same percentage of spontaneous alternations (no working memory differences). **P < 0.01.

Importantly, only select behaviors were affected by DAT-Cre KI. In a novel object preference test, we found no difference between heterozygous KI and WT mice in either the proportion of novel object visits (T-tests, *P* > 0.05 for all comparisons) or in novel object discrimination index (T-tests, *P* > 0.05 for all comparisons), regardless of sex (Fig. 3A), suggesting that novelty detection and memory is not affected in KI mice. Similarly, there was no genotype effect in either sex in any of the measured parameters on the hole board exploration test (Fig. 3B), including the number of head dips, duration of head dips, latency to first head dip or percentage of repeat head dips (Mann–Whitney tests, *P* > 0.05 for all comparisons). The fact we did not observe a genotype difference in the hole board test, together with the lack of a genotype or sex effect in the habituation dynamics in the open field (Fig. 1E), indicate that the observed KI effects on open field behavior are specific to locomotion, not exploratory drive. Finally, we tested spontaneous alternations in the plus maze and found that there was no difference between KI and WT mice of either sex in the proportion of spontaneous alternations (T-tests, *P* > 0.05 for all comparisons), demonstrating that working memory was not impaired in KI mice (Fig. 3C). Male KI mice did show a significant increase in the total number of arm entries during the test (T-test, *P* = 0.007**), which could be a reflection of a stronger constitutive hyperactivity phenotype already detected in the open field test, but no such difference was observed between female KI and WT mice (T-test, *P* = 0.273).

### DAT-Cre KI mice have reduced striatal DAT expression

**Figure 4:**
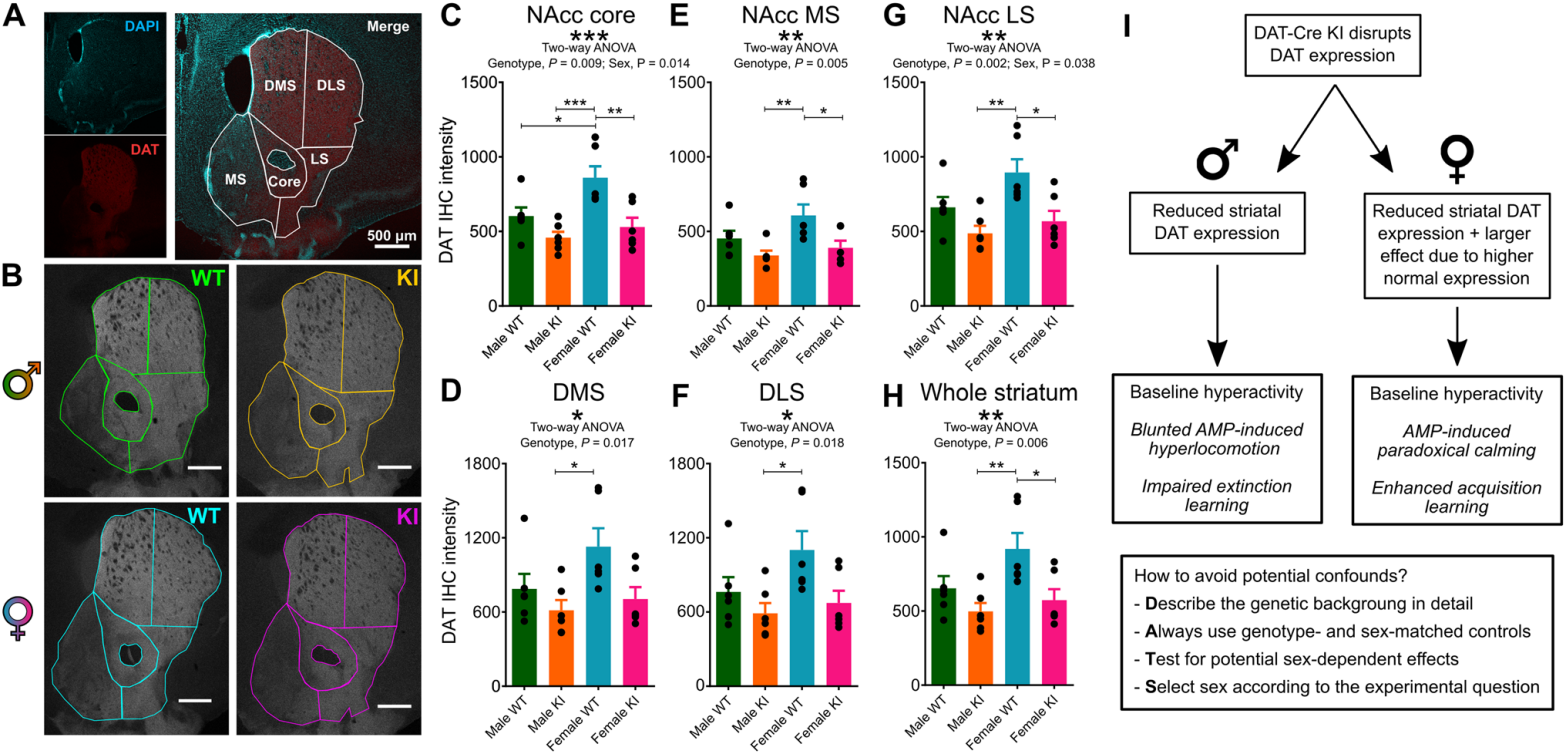
DAT-Cre KI mice have reduced striatal DAT expression, with females being disproportionately affected. (**A**) Representative maximum intensity projection of an analyzed striatal slice, indicating DAPI and DAT stainings and the striatal subregion division used throughout the analyses. NAcc = nucleus accumbens; MS = medial shell; LS = lateral shell; DMS = dorsomedial striatum; DLS = dorsolateral striatum. (**B**) Representative images of DAT immunostaining for each combination of sex and genotype. (**C**–**H**) Mean DAT IHC intensity in each striatal subregion for all experimental groups. Note that throughout the striatum there were significant effects of genotype on DAT expression. There was also a significant effect of sex in the NAcc core and LS subregions. According to Tukey’s post-hoc test, differences between groups were more prominent in the NAcc, with significant differences being confirmed between male and female WTs in the NAcc core (**C**), and between female KI and WT in all regions of the NAcc (**C**,**E**,**G**). In the DMS and DLS, only differences between male KI and female WT were confirmed in post-hoc tests (**D**,**F**). After averaging across all subregions, differences were confirmed between female WT and KI, and between male KI and female WT. (**I**) Schematic summarizing a potential mechanistic chain between the observed differences in DAT expression and in behavior, as well as recommendations to avoid potential confounds in light of the observed differences. *P < 0.05; **P < 0.01; ***P < 0.001.

What is the mechanism underlying these behavioral effects? Given that in the initial description of the line it was proposed that DAT-Cre KI mice might result in DAT haploinsufficiency^20^ and the profile of the behavioral changes we found approximated effects observed in mice with full or heterozygous DAT KO^21^, we surmised that our observed effects could be due to a reduction in striatal DAT expression. We tested this hypothesis using semi-quantitative immunohistochemistry (IHC) to measure DAT expression in different striatal regions of mice of both sexes and genotypes. A two-way ANOVA analysis revealed a significant genotype effect on DAT immunoreactivity across all areas of the striatum (*F*_1,20_ > 6.766 and *P* < 0.05 in all areas, Fig. 4A–H), with the highest effect being observed in the nucleus accumbens (NAcc) core (*F*_1,20_ = 15.2, *P* = 0.0009, Fig. 4C). Importantly, a significant effect of sex was also observed in the NAcc core (*F*_1,20_ = 7.271, *P* = 0.014, Fig. 4C) and the NAcc lateral shell (LS, *F*_1,20_ = 4.905, *P* = 0.038, Fig. 4G). Tukey post-hoc tests identified significant differences in DAT immunoreactivity between female WT and KI in all regions of the NAcc and in the sum of all striatal regions (*P* < 0.05, Fig. 4C,E,G,H), with the highest effect observed in the NAcc core (*P* = 0.005, Fig. 4C), but not in the DMS or DLS (*P* > 0.05, Fig. 4D,F). There were also significant differences in DAT levels between male KI and female WT mice in all striatal regions (*P* < 0.05), and in the NAcc core there was a significant difference between male WT and female WT (*P* < 0.05, Fig. 4C).

These findings demonstrate that female and male WT mice already have different DAT expression profiles, with females showing higher DAT immunoreactivity in the NAcc core and LS. Additionally, as predicted, KI mice of both sexes showed reduced DAT expression across the entire striatum. This effect was, however, larger in female KIs, given that female WTs already had higher baseline expression levels. It was also more pronounced in the NAcc subregions, especially the core, being less evident in the DMS and DLS. While levels of expression were similar for both male and female KIs, for female KIs this meant that they only had about half of the average expression of female WTs across the whole striatum, while male KIs were only showed on average a ~ 30% reduction in relation to male WTs.

## Discussion

We report that heterozygous DAT-Cre KI mice, widely used in neuroscience research for targeting dopamine neurons, display striking and sex-dependent behavioral differences in relation to WT littermates. Male KIs showed a ~ 45% increase in locomotor activity (Fig. 1C), a ~ 35% attenuation in responses to amphetamine (Fig. 1F), delayed extinction learning (~ 30% response difference in the first extinction session and no differences in acquisition learning (Fig. 2C). Female KIs exhibited a ~ 35% increase in locomotor activity (Fig. 1D) and did not display any amphetamine-induced hyperlocomotion; in fact, they exhibited a strong reduction in locomotor activity in response to an amphetamine dose that more than doubled this measure in female WTs (Fig. 1G,H). This translated to on average an eight-fold difference between the groups. Moreover, female KIs showed increased responding throughout acquisition learning, but normal extinction learning (Fig. 2D,F). By comparison, DAT-Cre KIs of both sexes had WT-like novelty preference learning, hole board exploration and working memory (Fig. 3).

We also found that DAT-Cre KIs showed reduced DAT expression in relation to WTs, which likely underlies the observed behavioral effects (Fig. 4). In support of this interpretation, similar behavioral phenotypes have been observed in mice with full or partial DAT KO, including hyperactivity^21–24^, blunted or paradoxical locomotor responses to amphetamine^21,25–27^, increased responding during acquisition of appetitive conditioning^28,29^ and resistance to extinction^30^ (but see also Rossi and Yin, 2015).

Our study adds to the growing number of reports characterizing inadvertent phenotypes of Cre-transgenic lines. Recently, similar behavior differences were also described for another DAT-Cre line, DAT^*IRES-cre*^, largely mirroring our findings^32^. In this line, heterozygous males have a trend of ~ 20% reduction in striatal DAT protein levels and males and females, when pooled together, have a 20–40% reduction in striatal dopamine reuptake^33,34^. This demonstrates that our findings may generalize to other DAT-Cre lines (but see also^35,36^). However, these studies did not look into interactions of these effects with sex, nor at the anatomical distribution of DAT expression changes.

Arguably, the most surprising finding in our study is that the observed alterations are categorically different between the two sexes. Our findings underscore the necessity for considering sex in behavioral-genetic studies, in line with the current NIH guidelines on considering sex as a biological variable^18^. They also fit with findings that fundamental neural processes can vary dramatically between the two sexes^16,17^.

Female KIs not only showed categorically different behavioral phenotypes in comparison to males, but also had a disproportionate reduction in DAT expression, given that female WTs showed higher DAT expression than male WTs, particularly in the NAcc core. It is likely that this is linked to the observed sex differences in behavioral phenotype (Fig. 4I), although it is unclear whether they are a direct consequence of striatal DAT reduction or of compensatory mechanisms. It is also not clear why WT females have higher DAT levels than males. This could be linked to other interacting physiological differences, which might include the enhanced functional coupling between D2 receptors and DAT observed in females (which is crucial, given that these receptors seem to mediate some behavioral effects of DAT KO), differential epigenetic regulation of dopamine-related genes and differences in the pre- and post-natal development of the midbrain dopamine system^16,29,37^. There are known differences in dopamine reuptake control, responses to amphetamines and DAT function between male and female rodents^37–39^. Furthermore, there are reports of sex differences in the behavioral and physiological effects of different DAT dysfunctions^40–43^.

The strongest difference we observed was in the response to amphetamine of female DAT-Cre KI mice, which not only did not display amphetamine-induced hyperlocomotion, but indeed reduced their activity in response to the stimulant. A similar phenomenon is observed in patients with attention deficit hyperactivity disorder (ADHD), which is why amphetamines are commonly prescribed to treat this disease^44^. This “paradoxical” or “calming” effect is also observed in mouse models of ADHD, where DAT function is genetically or pharmacologically impaired^45,46^. Our observation that female KIs had a higher reduction in DAT expression than males is consistent with a DAT hypofunction effect that would explain the sex-selective calming effect. In mice, DAT hypofunction typically shifts amphetamine dose–response relationships, with “paradoxical” calming being observed at low doses (2–10 mg/kg) and, if any, only blunted hyperlocomotion at higher doses^26,27^. While this was mostly demonstrated in male mice, in which we observed only a minor impact on amphetamine responses, a sex-dependent effect similar to what we observed has been reported in a social isolation mouse model of ADHD, in which maternally-deprived females showed paradoxical responses to low doses of amphetamine^47^. This indicates that the relationship between DAT dysfunction, sex and ADHD symptoms might be complex and depend on currently unrecognized factors. Our finding might thus be relevant to the understanding of ADHD pathophysiology, where there is a well-known gender difference in incidence and symptomatology^48^.

It is harder to explain the observed sex differences in reinforcement learning, mostly due to the paucity of studies characterizing how DAT hypofunction affects this process. Two previous studies found that male mice with partial DAT KO had faster acquisition of instrumental reward seeking^28,29^, and one study found that female DAT KO mice had normal acquisition but delayed extinction, also of instrumental reward seeking^30^. These results seem at odds with our observations, but the applied paradigms are not directly comparable with our task, which has a Pavlovian discriminative stimulus. It would seem that the effect on learning dynamics might depend on an interaction of task parameters, sex and the degree of DAT hypofunction. In the context of our task, the fact that differences were more prominent in the initial sessions of each phase of the task and tended to converge over time suggest differences in learning rate, rather than general motivation, hyperactivity or capacity to perform.

Importantly, our DAT-Cre line was maintained in a C57B/6N background. Effects of experimental mutations are influenced by genetic background^49–51^, including those that should be functionally neutral, such as the in the Drd2-EGFP mice, which overexpress D2 receptors when backcrossed to swiss mice, but not when maintained in C57BL/6 or FVB/N backgrounds^52–54^. It has also been specifically demonstrated that behavioral effects of DAT dysfunction vary between mice of different backgrounds. This includes an up to 13-fold difference in DAT KO-induced hyperlocomotion between mice of C57BL/6JOrl and DBA/2JOrl backgrounds^55^ and a shift in the dose–response curve for amphetamine in mice with a loss-of-function DAT mutation in a pure C57BL/6J background, compared to C57BL/6J-129Sv/J hybrids^27^. Furthermore, locomotor activity, amphetamine responses and appetitive conditioning vary according to mouse strain even without any induced mutagenesis^56–59^. Therefore, it is possible that there could be qualitative and quantitative differences between the effects reported here and those observed in DAT-Cre KI lines with different genetic backgrounds. The potential for genetic background-induced variability in the effects of DAT-Cre KI highlights the necessity of clear identification of mouse strains in publications^50,51^.

Based on our results, we propose that it is essential to test behavioral phenotypes of Cre-dependent mutations (e.g. dopamine neuron-specific conditional gene knock-outs, knock- downs or knock-ins) against sex-matched littermates with heterozygous DAT-Cre KI but lacking Cre-dependent gene expression (i.e. absence of floxed target sequences). Using littermate controls without the floxed gene, but not differentiating between full WT and heterozygous DAT-Cre carriers as controls, as has been done in studies using this strain^60–64^, as well as with the DAT^*IRES-cre*^ strain^65–71^, might mislead interpretations of experimental results.

Given our findings, we propose the following recommendations (Fig. 4I) for behavioral studies using DAT-Cre lines: (1) Describe the background strain in detail, including checking for potential genetic interactions of the experimental intervention with known characteristics of the strain; (2) Always use sex-matched littermates without Cre-dependent gene expression (or expressing a reporter protein) as controls; (3) Test for potential effects of sex, as even with matching there could unexpected interactions; (4) Select a particular sex according to the study objectives (for example, it would not be recommended to use female DAT-Cre KIs for investigating stimulant abuse). These practices could reduce potential confounds in the interpretation of results obtained with this important line.

## Methods

### Animals

All animal procedures described in this study were conducted in accordance with the guidelines of the German Animal Protection Act were approved by the German Regierungspräsidium Gießen and Darmstadt (license numbers: V54-19c 20/15 –FU/1100 & FU1203) and were carried out in compliance with the ARRIVE guidelines. Male and female heterozygous DAT-Cre KI (DAT-Cre^+/WT^) and WT (DAT-Cre^WT/WT^) littermate mice were bred and housed until 8 weeks of age at MFD diagnostics (Mainz, Germany). This particular line was established by backcrossing DAT-Cre mice (a.k.a. *Slc6a3*^*tm1(cre)Xz*^; Jackson Laboratory stock number: 020080;^20^) to the C57B/6N strain for over 6 generations. Littermates of both genotypes were obtained by crossing heterozygous DAT-Cre KIs with WT mice. Animals were then maintained under a 12 h dark/light cycle and housed in groups of two to four animals with food (R/M-keeping, Ssniff, Germany) and tap water available ad libitum, until the start of the experiments. Nesting material and a red acrylic glass shelter (mouse house, Tecniplast, Germany) were used as enrichment. Mice were individually housed for the duration of the experiments. KI and WT littermates were always tested in the same block of experiments. The experimenters were blind to the genotype of the mice for all performed procedures and data analysis; mouse order and group allocation was pseudo-randomized by a third party following a minimization strategy. The number of mice for each group was determined based on similar experiments from the literature.

### Spontaneous locomotor activity and amphetamine-induced hyperlocomotion

Spontaneous locomotor activity of KI (male N = 10; female N = 10) and WT (male N = 11; female N = 9) mice was assessed in an open field arena (a lidless box measuring 52 × 52 cm, under 3 lx of red light) using a video tracking system (Viewer II, Biobserve, Germany), as described previously^72^. The center of the arena was defined as a 30 × 30 cm square zone with all sides equidistant from the walls. Mice were tested for two days. In the first day, the animals were placed in the arena for 20 min (acclimation), then quickly removed, injected with saline (vehicle control; NaCl 0.9%, i.p.; Braun, Germany) and placed back in the arena for another 40 min (Fig. 1B). In the second day, this procedure was repeated, but instead of vehicle, the mice received a 2 mg/kg i.p. dose of d-amphetamine (Sigma-Aldrich, Germany), to evaluate the degree of amphetamine-induced hyperlocomotion^58^.

### Reinforcement learning task

KI (male N = 9; female N = 13) and WT (male N = 8; female N = 9) mice were tested in an appetitive conditioning task in which they learned that a conditioned stimulus (CS) signaled the availability of reward. For this task, mice were water restricted to ~ 85% of their initial body weight and were rewarded with a solution of 10% sucrose in tap water (reward). Daily water rations varied between 1 and 1.5 ml depending on the animal’s weight on that day, with a set target of 85% of the initial body weight. Except during experimental sessions, water was always delivered in a cup placed in their home cage. Mice were also closely monitored in order to ensure that the water supply was consumed and not spilt over or contaminated and their health status was evaluated daily^73^.

The full experimental paradigm spanned 24 days. On the first four days, mice were submitted only to water restriction. In the following two days, they were handled until they no longer tried to escape from the experimenter’s hand, showed no overt signs of stress and anxiety and readily drank a portion of liquid reward (0.2 ml) given by the experimenter via a syringe while being held. The following day, animals where placed inside an empty operant chamber for 50 min (acclimation). The day after that, mice underwent shaping, i.e. they were placed in the operant chamber for another 50 min, now with a reward port (liquid cup) present, and at semi-random time intervals (mean of 60 s, 30 to 90 s range), a reward portion (16.7 µl) was delivered at the port. Port entries were detected by the breaking on an infra-red beam.

The following day, mice started the conditioning task^74^. Animals learned to associate the CS (sound tone pulsed at 3 Hz—0.1 s on/0.2 s off—at 70 dB) with the availability of reward (acquisition; Fig. 2B). Each session consisted of ten trials (mean ITI of 4 min, 1.5 to 6.5 min range) in which the auditory cue was on for 30 s. Animals could trigger reward delivery to the port by entering it during CS presentation. Rewards were delivered in a cycle of 2 s reward delivery (16.7 µl) followed by a 3 s consumption interval. Delivery was continuous for as long as the animal kept its head in the port during CS presentation. This allowed for a maximum of 6 rewards per trial and a maximum of 60 rewards per session (a total of ~ 1 ml or reward in the task per day). Additional water supplementation, when needed to complete the animal’s daily water ration, was provided in the home cage. This acquisition phase lasted for 11 daily sessions. Immediately a day after the acquisition ceased, extinction of the conditioned response was tested by omitting rewards for six daily sessions (Fig. 2B). After extinction, animals were returned to their home cage and received water ad libitum for at least three days before being submitted to the other behavioral tasks listed in the following subsections (novel object preference, hole board, spontaneous alternation). All animals recovered their initial body weight in this period and showed no long-term adverse effects from water restriction.

Performance in this task was quantified by the total time the animals spent in the port during the cued trials and the latency to enter the port after cue onset. Time in port was normalized both as a percentage of total reward availability time and also by subtracting the amount of time the animal spend in the port 30 s before cue onset^74^. Latency was quantified as the time between cue initiation and the first head entry into the reward port; if animals did not respond in a trial, the maximal possible latency value (30 s) was ascribed to that trial.

### Novel object recognition

Novel object exploration was assessed in the same arena and with the same software as the open field test (Fig. 3A). Mice were placed in the arena for 10 min (acclimation), after which they were placed back in their home cages and two identical objects (stainless steel cylinders; 3 cm diameter × 6 cm height) were placed in the arena at equal lengths from each other and at 15 cm from the upper left and right corners. The animals were returned to the arena and allowed to freely explore the objects for 10 min (trial 1), after which they were again removed from the box. Subsequently, one of the objects was replaced by a different, novel object (plastic coated rectangular prism; 3 × 3 cm base × 6 cm height), and the animals were again allowed to explore the arena and the objects (trial 2)^75,76^. Mice were placed in their home cage in between trials only for the time necessary to place or replace the objects.

Object recognition was analyzed using Biobserve’s Object Recognition plug-in, and object interaction events were defined as periods in which a mouse was directly facing the object (snout directed to the object within a 180° angle) at a distance shorter than 3 cm. Both the number and duration of these interaction events were quantified for both trials of the task. Exploration dynamics (number and duration of objected-directed exploration) were analyzed for both trials. Novel object preference for each group was quantified by the percentage of visits to the novel object on trial 2 (out of all object visits) and object exploration discrimination index, which is calculated by dividing the difference between the total time spent exploring the novel object and the time spent exploring the familiar object by the sum of these two measures^75,76^:

$$DI= \frac{{T}_{New}-{T}_{Fam}}{{T}_{New}+{T}_{Fam}}$$

where $DI$ = discrimination index, ${T}_{New}$ = time spent exploring the novel object, ${T}_{Fam}$ = time spent exploring the familiar object,

### Hole board

Spontaneous (unrewarded) exploratory activity was evaluated with the hole board task^77,78^. Mice were placed for five minutes in an open arena (50 × 50 cm) with 4 circular holes (2 cm in diameter) on its floor (Fig. 3B). Head dips were recorded as breaks in infra-red beams placed immediately under the inferior surface of the floor board (Actimot2, TSE systems). The latency to the first head dip, as well as the number and duration of head dips, were quantified. The percentage of repeated head dips (two dips performed sequentially into the same hole) was also quantified and compared between genotypes.

### Spontaneous alternation in the plus maze

Working memory performance was quantified between genotypes using the spontaneous alternation in the plus maze^79^. Mice were placed in a plastic plus maze (four equidistant 35 × 4.5 cm arms radiating at 90° angles from a circular central arena with 10 cm diameter; walls were 15 cm in height; Fig. 3C) and allowed free exploration for 12 min^80^. An arm entry was scored when the animal entered an arm with all its four paws. A spontaneous alternation was marked when the animal explored four different arms in five consecutive arm entries, and the proportion of spontaneous alternations was quantified as the number of real alternations divided by the total possible number of alternations (sum of all arm entries minus four); chance performance in this task is calculated to be 44%^79,80^.

### Semi-quantitative immunohistochemistry

Six mice of each sex and genotype combination (male and female, KI and WT) were injected with a lethal dose (1.6 g/kg) of sodium pentobarbital and intracardially perfused with and an ice cold solution of 4% paraformaldehyde (PFA) and 15% picric acid in phosphate buffered saline (PBS; 137 mM NaCl, 2.7 mM KCl, 10 mM NaH_2_PO_4_, 10 mM Na_2_HPO_4_; pH = 7.4). Each brain was subsequently removed, post-fixed for 24 h in the PFA solution, and then transferred to a sucrose storing solution (0.01 M PBS, 10% sucrose, 0.05% NaN3) until sectioning. Coronal 50 µm brain sections were made with a microtome (VT1000S, Leica, Germany). Sections were washed in PBS and then incubated in blocking solution (0.01 M PBS, 10% horse serum, 0.5% Triton X-100, 0.2% bovine serum albumin—BSA) for one hour. After blocking, sections were incubated overnight at room temperature in carrier solution (0.01 M PBS, 1% horse serum, 0.5% Triton X-100, 0.2% BSA) with 1:1000 anti-DAT monoclonal rat primary antibody (catalog no. MAB369, Merck Millipore, USA). Sections were again washed three times in PBS and incubated for at least six hours in carrier solution with 1:750 goat anti-rat AlexaFluor® 568 secondary antibody (catalog no. A11077, Invitrogen, USA). Sections were washed three more times in PBS, incubated with 0.2 µl/ml DAPI (catalog no. D1306, Invitrogen, USA) for 5 min and then washed two times in PBS. Sections were then placed on glass microscope slides (76 × 26 mm, Menzel, Germany), covered with Vectashield® mounting medium and glass coverslips, sealed with nail polish and stored at + 4 °C until visualization. A laser-scanning confocal microscope (Eclipse 90i, Nikon, Japan) was used to acquire Z-stacks of striatal DAT IHC stainings, with the same settings being applied to all slices. Analysis of the acquired images was performed using ImageJ. First, one representative Z-stack was selected around the same AP coordinates for each mouse. The DAT-IHC channel of the image was then collapsed into a maximum intensity projection and custom ROIs were delineated around individual striatal subregions based on anatomical landmarks defined in the mouse brain atlas (Franklin and Paxinos, 2012). The average intensity for each ROI was then measured and saved for analysis.

### Statistical analyses

For all two-group comparisons, data were tested for normality with the Kolmogorov–Smirnov test. If both distributions were Gaussian, differences were analyzed using unpaired two-tailed T-tests; otherwise, the two-tailed Mann–Whitney test was used. Comparisons between genotype groups over multiple time points or sessions were performed using two-way repeated measures ANOVA and the Sidak’s multiple comparisons post-hoc test. Comparisons between genotype and sex groups for DAT immunoreactivity experiments were analyzed using regular two-way ANOVA with Tukey’s post-hoc test. All statistical analyses were done in GraphPad Prism 8.0 (GraphPad, USA). Statistical significance was set at *P* < 0.05 for all comparisons. Data points and error bars in graphs represent means and standard errors from the mean; overlaid lines and dots represent individual data from each subject.

## Data Availability

The datasets generated during and/or analyzed during the current study are available from the corresponding author on reasonable request.
